# Supplementary material for: Association between Proteinuria Status and Risk of Hypertension: A Nationwide Population-Based Cohort Study
Source: J Pers Med. 2023 Sep 20;13(9):1414. doi: 10.3390/jpm13091414 (PMC10533010; doi:10.3390/jpm13091414)
Supplement: Supplementary file 1 [file jpm-13-01414-s001.zip › jpm-2529752-supplementary.pdf]

Supplementary materials

Table S1. Multivariable Cox analysis for incident hypertension according to degree of proteinuria.

Degree of proteinuria	Total (n)	Hypertension (n)	Incidence rate (per 1000 person years)	HR (95% CI)		
				**Model 1**	**Model 2**	**Model 3**
Negative	926532	342391	31.55	1 (ref)	1 (ref)	1 (ref)
1+	6762	3060	42.41	1.35 (1.30,1.39)	1.32 (1.27, 1.36)	1.31 (1.27, 1.36)
2+	2028	1020	50.04	1.59 (1.50,1.69)	1.55 (1.46, 1.65)	1.55 (1.46, 1.65)
3+	330	179	58.04	1.85 (1.60,2.14)	1.85 (1.601, 2.15)	1.86 (1.61, 2.16)
4+	71	36	55.15	1.76 (1.27,2.44)	1.81 (1.31, 2.52)	1.80 (1.30, 2.50)
		p for trend		<0.001	<0.001	<0.001

Model 1 was adjusted for age and sex.

Model 2 was adjusted for age, sex, body mass index, household income, smoking, alcohol consumption, physical activity, history of diabetes mellitus, dyslipidemia, atrial fibrillation, cancer, and renal disease.

Model 3 was adjusted for age, sex, body mass index, household income, smoking, alcohol consumption, physical activity, history of diabetes mellitus, dyslipidemia, atrial fibrillation, cancer, renal disease, and Charlson Comorbidity Index.

HR, hazard ratio; CI, confidence interval;

Table S2. Multivariable Cox analysis for incident hypertension according to changes in proteinuria status (landmark analysis).

Group	Total (n)	Hypertension (n)	Incidence rate (per 1000 person-years)	HR (95% Confidence Interval)		
				Model 1	Model 2	Model 3
Proteinuria-free	919044	337193	31.1	1 (ref)	1 (ref)	1 (ref)
Proteinuria-recovered	7488	3120	37.0	1.19 (1.15, 1.24)	1.17 (1.13, 1.22)	1.17 (1.13, 1.21)
Proteinuria-developed	8205	3605	40.5	1.31 (1.26, 1.35)	1.30 (1.26, 1.35)	1.31 (1.26, 1.35)
Chronic proteinuria	986	658	79.6	2.62 (2.42, 2.82)	2.12 (1.96, 2.29)	2.09 (1.94, 2.26)
		p for trend		<0.001	<0.001	<0.001

Landmark analysis was performed excluding those who had developed hypertension within one year from the index date.

Model 1 was adjusted for age and sex.

Model 2 was adjusted for age, sex, body mass index, household income, smoking, alcohol consumption, physical activity, history of diabetes mellitus, dyslipidemia, atrial fibrillation, cancer, and renal disease.

Model 3 was adjusted for age, sex, body mass index, household income, smoking, alcohol consumption, physical activity, history of diabetes mellitus, dyslipidemia, atrial fibrillation, cancer, renal disease, and Charlson Comorbidity Index.

HR, hazard ratio; CI, confidence interval;

Table S3. Pairwise comparisons of the association between change in proteinuria status and risk of incident hypertension (landmark analysis).

	Adjusted HR (95% CI)*	P value
Proteinuria-resolved vs. Proteinuria-free (reference)	1.15 (1.12, 1.90)	<0.001
Proteinuria-developed vs. Proteinuria-free (reference)	1.31 (1.26, 1.36)	<0.001
Proteinuria-resolved vs. Chronic proteinuria (reference)	0.64 (0.59, 0.69)	<0.001
Proteinuria-developed vs. Chronic proteinuria (reference)	0.75 (0.69, 0.80)	<0.001

Landmark analysis was performed excluding those who had developed hypertension within one year from the index date.

*Adjusted for age, sex, body mass index, household income, smoking, alcohol consumption, physical activity, history of diabetes mellitus, dyslipidemia, atrial fibrillation, cancer, renal disease, and Charlson Comorbidity Index.

HR, hazard ratio; CI, confidence interval;

Table S4. Multivariable Cox analysis for incident hypertension according to changes in proteinuria status (sensitivity analysis).

Group	Total (n)	Hypertension (n)	Incidence rate (per 1000 person-years)	HR (95% Confidence Interval)		
				Model 1	Model 2	Model 3
Proteinuria-free	221931	70761	26.0	1 (ref)	1 (ref)	1 (ref)
Proteinuria-recovered	1841	656	29.9	1.20 (1.11, 1.29)	1.17 (1.09, 1.27)	1.17 (1.08, 1.26)
Proteinuria-developed	1987	735	31.8	1.29 (1.20, 1.39)	1.28 (1.19, 1.37)	1.27 (1.18, 1.37)
Chronic proteinuria	216	128	62.1	2.33 (1.96, 2.76)	2.21 (1.86, 2.63)	2.20 (1.85, 2.62)
		p for trend		<0.001	<0.001	<0.001

Sensitivity analysis was performed excluding the participants who met any of the following criteria: over 65 years old, more than 25 (kg/m^2) of BMI, current smoker, those without regular exercise, and those with a history of renal disease.

Model 1 was adjusted for age and sex.
Model 2 was adjusted for age, sex, household income, alcohol consumption, history of diabetes mellitus, dyslipidemia, atrial fibrillation, and cancer.
Model 3 was adjusted for age, sex, household income, alcohol consumption, history of diabetes mellitus, dyslipidemia, atrial fibrillation, cancer, and Charlson Comorbidity Index.
HR, hazard ratio; CI, confidence interval.

Table S5. Pairwise comparisons of the association between change in proteinuria status and risk of incident hypertension (sensitivity analysis).

	**Adjusted HR (95% CI)***	**P value**
Proteinuria-resolved vs. Proteinuria-free (reference)	1.14 (1.12, 1.16)	<0.001
Proteinuria-developed vs. Proteinuria-free (reference)	1.25 (1.21, 1.29)	<0.001
Proteinuria-resolved vs. Chronic proteinuria (reference)	0.84 (0.76, 0.92)	<0.001
Proteinuria-developed vs. Chronic proteinuria (reference)	0.79 (0.73, 0.85)	<0.001

Sensitivity analysis was performed excluding the participants who met any of the following criteria: over 65 years old, more than 25 (kg/m^2) of BMI, current smoker, those without regular exercise, and those with a history of renal disease.

*Adjusted for age, sex, body mass index, household income, smoking, alcohol consumption, physical activity, history of diabetes mellitus, dyslipidemia, atrial fibrillation, cancer, renal disease, and Charlson Comorbidity Index.

HR, hazard ratio; CI, confidence interval.
